# Sestrin2 and sestrin3 suppress NK-92 cell-mediated cytotoxic activity on ovarian cancer cells through AMPK and mTORC1 signaling

**DOI:** 10.18632/oncotarget.21487

**Published:** 2017-10-04

**Authors:** Xuejin Wang, Weifeng Liu, Deyi Zhuang, Shaoxian Hong, Jingfang Chen

**Affiliations:** ^1^ Department of Reproductive Medicine, The Second Affiliated Hospital of Fujian Medical University, Quanzhou 362000, China; ^2^ Department of Anesthesiology, The Second Affiliated Hospital of Fujian Medical University, Quanzhou 362000, China; ^3^ Department of Neonatal, Children's Hospital of Fudan University Xiamen Branch (Xiamen Children's Hospital), Xiamen 361000, China; ^4^ Department of Pediatric Intensive Care Unit, Children's Hospital of Fudan University Xiamen Branch (Xiamen Children's Hospital), Xiamen 361000, China; ^5^ Department of Pediatrics, Children's Hospital of Fudan University Xiamen Branch (Xiamen Children's Hospital), Xiamen 361000, China

**Keywords:** NK cells, ovarian cancer, sestrin, mTOR, AMPK

## Abstract

Ovarian cancer is one of the major cancer types. NK-92 cell line, which has consistently and reproducibly high anti-tumor cytotoxicity, may be used for immunotherapy against ovarian cancer. Understanding the mechanisms that regulate the anti-tumor activity of NK-92 cells is important for developing novel therapeutic strategies. In the current study, using an ovarian cancer xenograft mouse model, we identified the up-regulation of sestrin2 (SESN2) and sestrin3 (SESN3) in intratumoral NK-92 cells. Lentivirus-transduced NK-92 cells, which overexpressed SESN2 or SESN3 after doxycycline treatment, exhibited less expression of activating receptors, perforin and granzyme B. Overexpression of SESN2 and SESN3 impaired tumoricidal effect of NK-92 cells both *in vitro* and *in vivo*. Furthermore, overexpression of SESN2 and SESN3 inhibited mTORC1 signaling while promoting AMPK signaling in NK-92 cells. Taken together, our data highlights the crucial effects of SESN2 and SESN3 on NK-92 cell-mediated anti-ovarian cancer activity. This study might be valuable for designing a novel therapeutic strategy for ovarian cancer.

## INTRODUCTION

Ovarian cancer is the one of the most common cancer in women all over the world. Although surgery and chemotherapy are available, the 5-year survival rate is still relatively low. Immunotherapy could be a promising therapeutic approach since ovarian cancer is an immunogenic tumor that can be recognized by the host immune system [1]. Recently NK-92 cell line has shown promising tumoricidal potency and therefore has undergone intensive study [2]. NK-92 cell line is easy to be genetically modified to distinguish certain tumor antigens or to promote the activity of monoclonal antibodies via antibody-dependent cytotoxicity [3]. Currently it is the only NK cell line which has undergoing clinical trials [4–6]. In addition, NK-92 cell line has been shown to kill ovarian cancer cells including OVCAR-3 [7–9]. However, the molecular mechanisms by which NK-92 cell activity is regulated in ovarian cancer remains unclear. NK cells might reduce or even lose their cytotoxic activity against tumor cells in some tumor patients [10, 11], due to altered expression of activating receptors and inhibitory receptors.

Sestrins (SESNs) are a family of highly conserved proteins that are induced upon various conditions of stress, including DNA damage and oxidative stress [12]. Their functions have not been well documented. SESN1 and SESN2 are intracellular leucine sensors and p53 target genes that restrain mTORC1 signaling [13, 14]. SESN3 enhances hepatic insulin sensitivity through activation of mTORC2-Akt signaling [15], and it also inhibits mTORC1 [16]. Due to the importance of mTORC1 for immune cell fucntions [17, 18], it is likely that SESNs impact immune reactions. Indeed, SESNs inhibit T cell immunity [19], maintain macrophage survival and inhibit inflammatory response [20, 21]. However, the significance of SESNs for NK cell development and function has not been addressed.

In this study, using an ovarian cancer xenograft mouse model and NK-92 cell line, we identified the up-regulation of SESN2 and SESN3 in intratumoral NK-92 cells. To determine the roles of SESN2 and SESN3 in the regulation of NK-92 cell activity, we established a lentivirus-mediated SESN inducible expression system in NK-92 cells. Lentivirus-transduced NK-92 cells, which expressed SESN2 or SESN3 upon doxycycline treatment, down-regulated expression of NKG2D and NKp44, perforin and granzyme B, and their tumoricidal activity was subsequently impaired both *in vitro* and *in vivo*. Furthermore, overexpression of SESN2 and SESN3 inhibited mTORC1 signaling while promoting AMPK signaling in NK-92 cells. Taken together, our data highlights the crucial effects of SESN2 and SESN3 on NK-92 cell-mediated anti-ovarian cancer activity.

## RESULTS

### Intratumoral NK-92 cells up-regulate SESN2 and SESN3 expression

**Figure 1:**
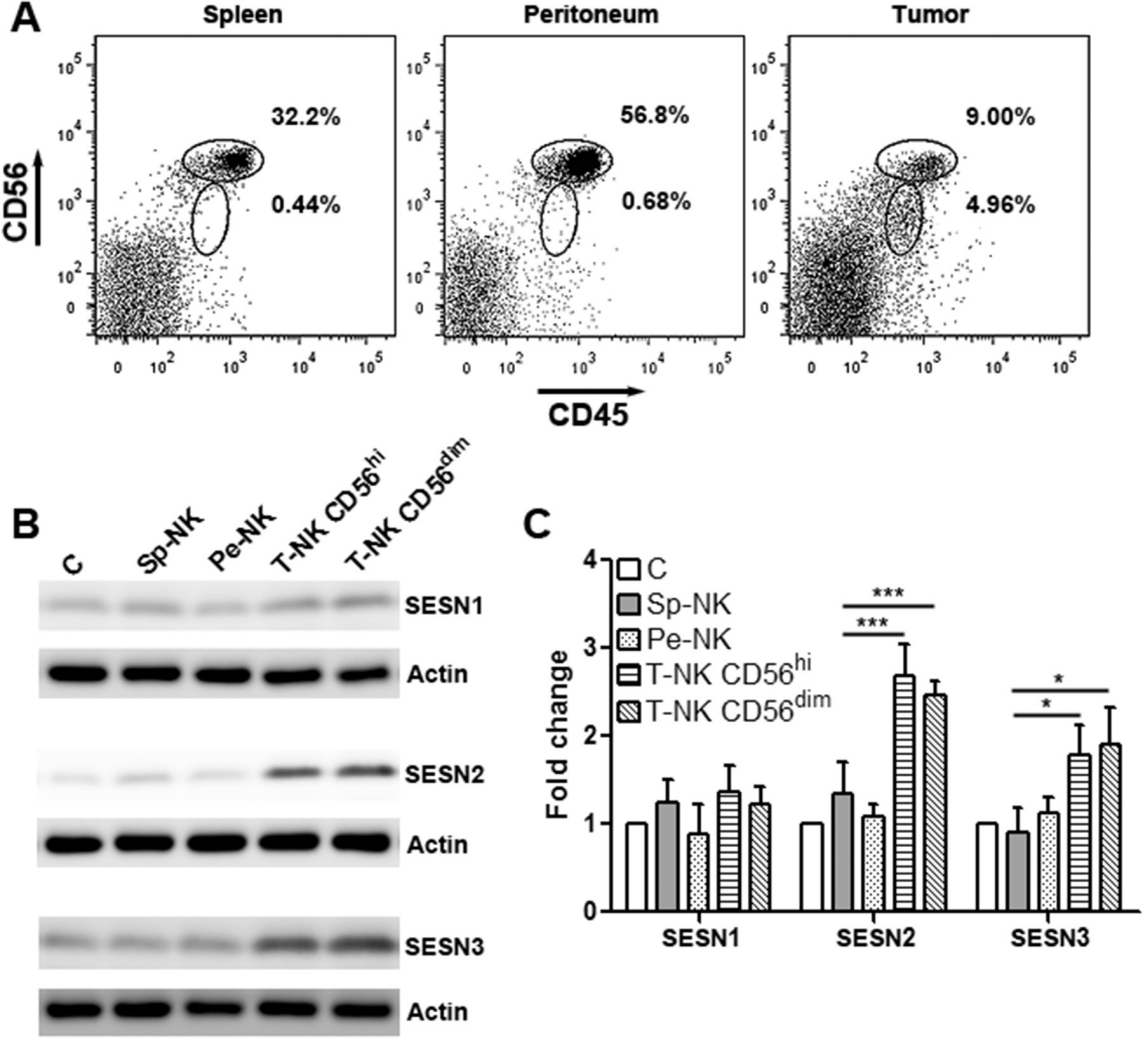
Intratumoral NK-92 cells up-regulate SESN2 and SESN3 expression **(A)** Representative flow cytometry plots showing CD45^+^CD56^+^ NK-92 cells in the spleens, peritoneal cavity and tumor xenografts. NK-92 cells were adoptively transferred into tumor-bearing mice through intraperitoneal injection and were then isolated from indicated tissues based on CD45 and CD56 expression. **(B)** SESN1, SESN2 and SESN3 protein levels in NK-92 populations isolated from different tissues. C: *in vitro* cultured NK-92 cells. Sp-NK: NK-92 cells isolated from spleens. Pe-NK: NK-92 cells isolated from peritoneal cavity. T-NK C56^hi^: intratumoral NK-92 cells expressing high CD56. T-NK CD56^dim^: intratumoral NK-92 cells expressing dim CD56. **(C)** Statistics for (B). N= 5 per group. ^*^, p<0.05; ^***^, p<0.001.

We firstly tested NK-92 cell activity in a subcutaneous OVCAR-3 xenograft model. CD45^+^CD56^+^ NK-92 cells were sorted from the spleen, peritoneum and tumor grafts of the tumor-bearing mice using flow cytometry. Notably, intratumoral NK-92 cells were divided into 2 subgroups according to their CD56 expression profile: CD56^hi^ and CD56^dim^, while splenic and peritoneal NK-92 cells were uniformly CD56^hi^ (Figure 1A). Although we did not observe a significant change in tumor volume (data not shown), we unintentionally found higher expression of SESN2 and SESN3 in intratumoral NK-92 cells than those in splenic and peritoneal NK-92 cells (Supplementary Figure 1 and Figure 1B). CD56^hi^ and CD56^dim^intratumoral NK-92 cells had equivalent expression of SESN2 and SESN3, whereas SESN1 expression was not dramatically changed (Figure 1B & 1C). In addition, expression of SESN2 and SESN3 in normal human blood NK cells were extremely low (Supplementary Figure 1). The up-regulation of SESN2 and SESN3 in intratumoral NK-92 cells was also observed in another subcutaneous ovarian cancer model using SKOV3 cells (Supplementary Figure 2).

### SESN2 and SESN3 suppresses NK-92 cell activation

**Figure 2:**
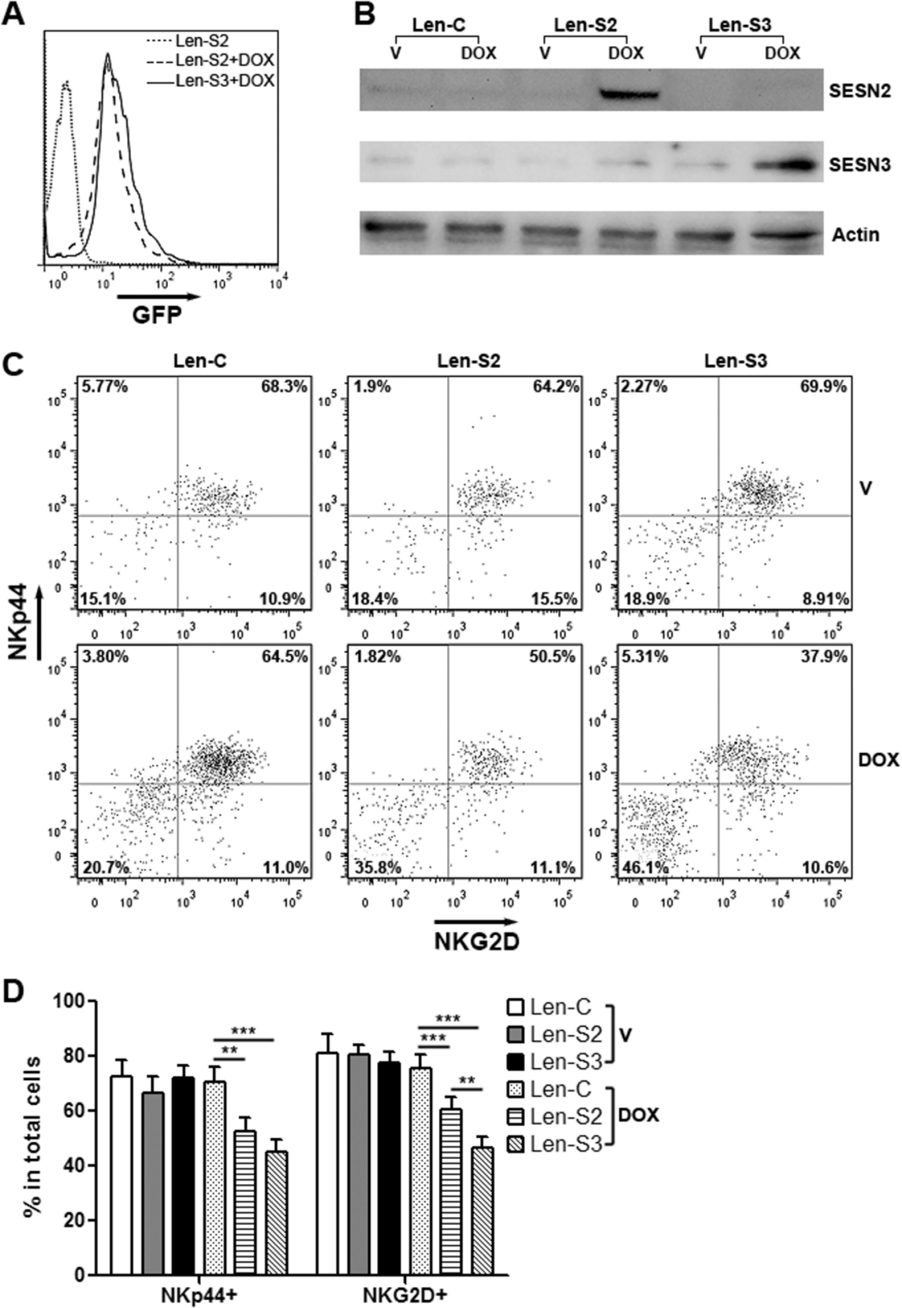
SESN2 and SESN3 expression inhibits NK-92 cell activation *in vitro* **(A)** GFP expression in lentivirus-transduced NK-92 cells with or without doxycycline treatment for 48 h. Len-S2: NK-92 cells transduced with Len-S2. Len-S3: NK-92 cells transduced with Len-S3. DOX: 200 ng/ml doxycycline. This is a representative image of three independent experiments. **(B)** SESN2 and SESN3 protein levels in doxycycline-treated NK-92 cells. Len-C: NK-92 cells transduced with lentivirus containing no SESN sequence. Len-S2: NK-92 cells transduced with Len-S2. Len-S3: NK-92 cells transduced with Len-S3. This is a representative image of two independent experiments. **(C** & **D)** Expression of NKp44 and NKG2D on NK-92 cells after treatment with vehicle (PBS) or doxycycline. Representative dot plots were shown in (C). Statistics was shown in (D). V: vehicle. DOX: doxycycline. N=6~8 per group. ^*^, p<0.05; ^**^, p<0.001; ^***^, p<0.001.

To explore the effects of SESN2 and SESN3, we established NK-92 cell lines expressing inducible SESN2 and SESN3, respectively (Supplementary Figure 3). Treatment with doxycycline remarkably induced expression of GFP and SESNs in these cells (Figure 2A & 2B). In addition, expression of two NK cell activating receptors, NKp44 and NKG2D, was significantly decreased on SESN2 or SESN3-overexpressing NK-92 cells (Figure 2C & 2D). Expression of other two NK activating receptors, Nkp30 and NKp46, were not profoundly changed (Supplementary Figure 4). The cell viability was not impacted by overexpression of SESN2 or SESN3 (Supplementary Figure 5).

### SESN2 and SESN3 suppresses cytotoxic activity of NK-92 cells *in vitro*

**Figure 3:**
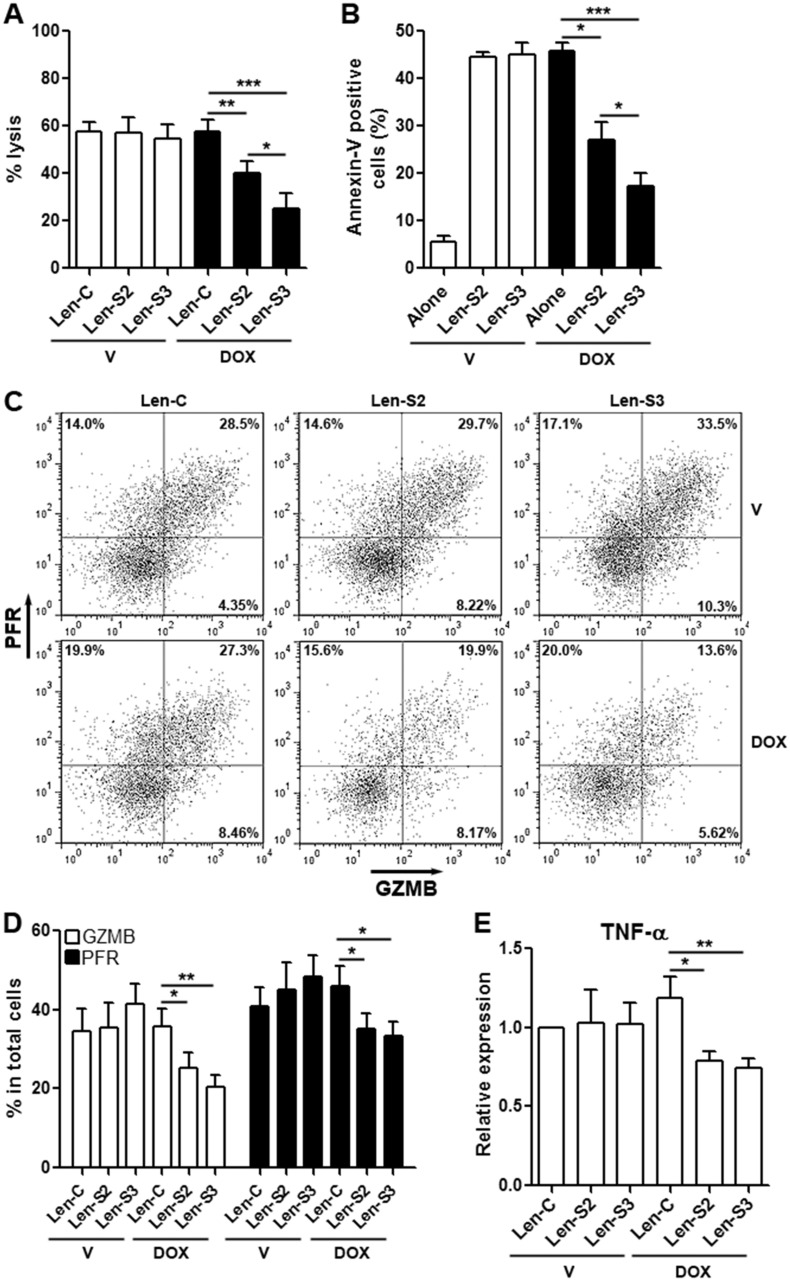
SESN2 and SESN3 expression suppresses NK-92 cell cytotoxic activity *in vitro* **(A)** OVCAR-3 cell lysis after co-culture with NK-92 cells. Lenitvirus-transduced NK-92 cells were pre-treated with vehicle or doxycycline for 48 h prior to co-culture with OVCAR-3 cells. V: vehicle. DOX: doxycycline. Len-C: NK-92 cells transduced with lentivirus containing no SESN sequence. Len-S2: NK-92 cells transduced with Len-S2. Len-S3: NK-92 cells transduced with Len-S3. N=6 per group. **(B)** OVCAR-3 cell apoptosis after co-culture with NK-92 cells. OVCAR-3 cells were distinguished from NK-92 cells via CD56 staining (Supplementary Figure 6). N=8 per group. **(C** & **D)** Expression of perforin (PFR) and granzyme B (GZMB) in lentivirus-transduced NK-92 cells after vehicle or doxycycline treatment. Representative dot plots were shown in (C), and statistics was shown in (D). N=5 per group. **(E)** Relative mRNA abundance of TNF-α in lentivirus-transduced NK-92 cells. N=3 per group. ^*^, p<0.05; ^**^, p<0.001; ^***^, p<0.001.

Lentivirus-transduced NK-92 cells were co-cultured with OVCAR-3 cells to evaluate cytotoxicity. SESN2 or SESN3-overexpressing NK-92 cells induced less lysis and apoptosis of OVCAR-3 cells, as compared with NK-92 cells not expressing SESNs (Figure 3A & 3B). Furthermore, expression of perforin and granzyme B, which are cytotoxic mediators, were profoundly reduced in SESN2 or SESN3-overexpressing NK-92 cells, in comparison with other groups (Figure 3C & 3D). Another cytotoxic factor, TNF-α, was moderately decreased in SESN2 or SESN3-overexpressing NK-92 cells (Figure 3E).

### SESN2 and SESN3 inhibits mTORC1 signaling and promotes AMPK signaling

**Figure 4:**
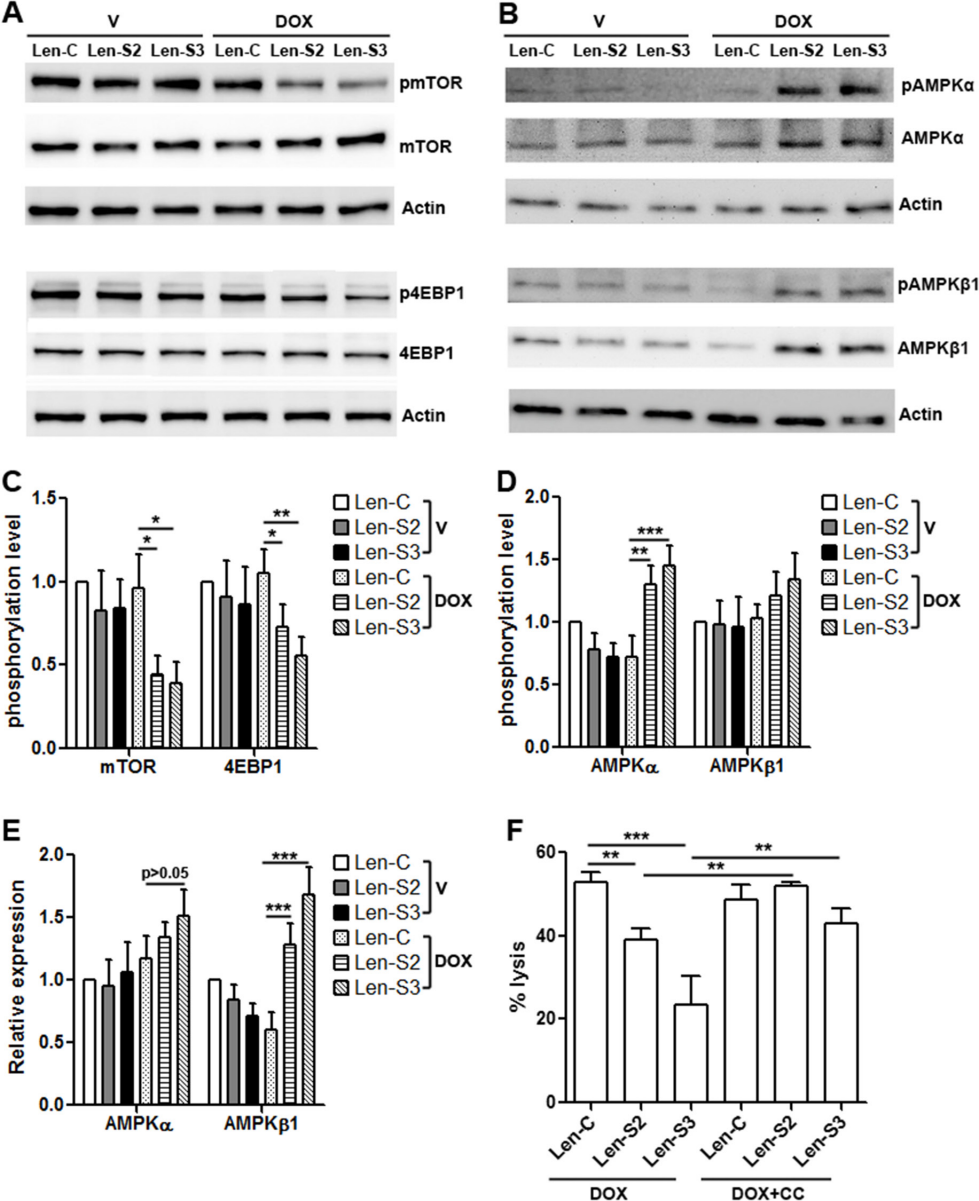
SESN2 and SESN3 expression inhibits mTORC1 signaling and promotes AMPK signaling **(A)** Representative Western blot images of phosphorylation of mTOR and 4EBP1 in lentivirus-transduced NK-92 cells. **(B)** Representative Western blot images of phosphorylation of AMPKα and AMPKβ1 in lentivirus-transduced NK-92 cells. **(C)** Statistics for phosphorylation of mTOR and 4EBP1. N=4 per group. **(D)** Statistics for phosphorylation of AMPKα and AMPKβ1. N=3 per group. **(E)** Statistics for total AMPKα and AMPKβ1 proteins. N=3 per group. **(F)** OVCAR-3 cell lysis after co-culture with NK-92 cells. Lenitvirus-transduced NK-92 cells were pre-treated with doxycycline and compound C before co-culture with OVCAR-3 cells. DOX: doxycycline. DOX+CC: doxycycline with compound C. N=8 per group. ^*^, p<0.05; ^**^, p<0.001; ^***^, p<0.001.

SESNs can suppress mTORC1 signaling [16, 22, 23]. To evaluate if this happened in NK-92 cells, activating phosphorylation of mTOR and 4EBP1 was determined. As shown in Figure 4A & 4C, after doxycycline treatment, phosphorylation of mTOR and 4EBP1 was remarkably decreased in SESN2 or SESN3-overexpressing NK-92 cells, while the protein levels of mTOR and 4EBP1 were not changed. Since AMPK signaling suppresses mTORC1 signaling [24], and SESNs can enhance AMPK signaling [25, 26], we checked AMPK activation and found that SESN2 or SESN3-overexpressing NK-92 cells expressed more phosphorylated AMPKα and AMPKβ1, in comparison with other groups (Figure 4B & 4D). Interestingly, the total AMPKβ1 protein was increased in SESN2 or SESN3-overexpressing NK-92 cells (Figure 4E). To determine the effect of AMPK signaling on cytotoxic activity of NK-92 cells, SESN2 or SESN3-overexpressing NK-92 cells were treated with AMPK inhibitor Compound C before co-culture with OVCAR-3 cells. Compound C inhibited AMPKα activation and promoted mTORC1 signaling (Supplementary Figure 7), and subsequently restored cytotoxic activity of SESN2 or SESN3-overexpressing NK-92 cells (Figure 4F).

### SESN2 and SESN3 suppresses NK-92 cell activity *in vivo*

**Figure 5:**
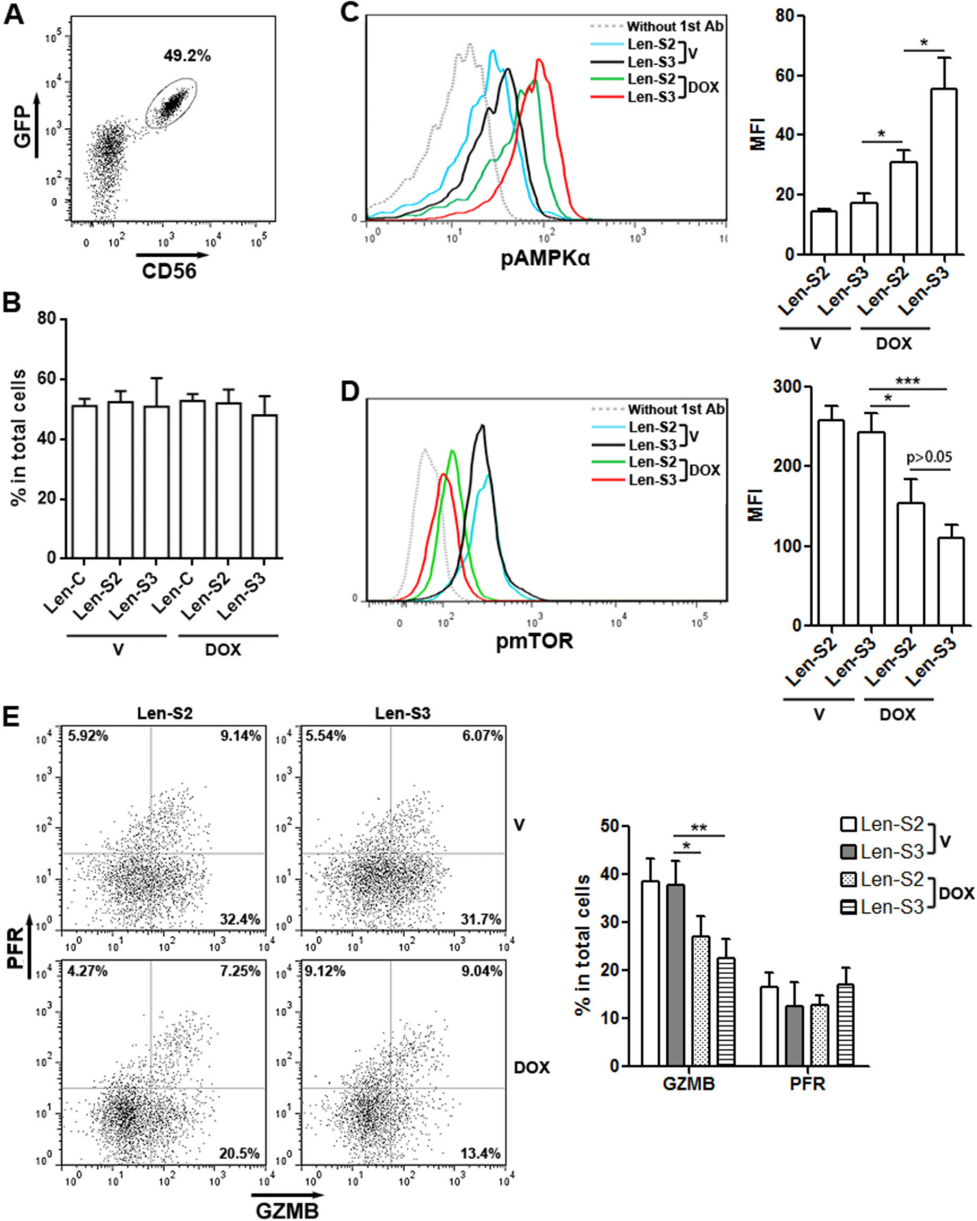
SESN2 and SESN3 inhibit NK-92 cell activity *in vivo* **(A)** Representative dot plot showing the expression of GFP and CD56 in Len-S2-transduced NK-92 cells isolated from peritoneal cavity after doxycycline pre-treatment and adoptive transfer. **(B)** Proportions of SESN2 or SESN3-overexpressing NK-92 cells and control NK-92 cells in total peritoneal cells on day 2 after adoptive transfer. V: vehicle. DOX: doxycycline. Len-C: NK-92 cells transduced with lentivirus containing no SESN sequence. Len-S2: NK-92 cells transduced with Len-S2. Len-S3: NK-92 cells transduced with Len-S3. N=3 per group. **(C** & **D)** Phosphorylated AMPKα (C) and mTOR (D) in transferred NK-92 cells was detected using flow cytometry. Left panels: representative histograms. Right panels: statistics. N=4 per group. **(E)** Expression of perforin (PFR) and granzyme B (GZMB) in transferred NK-92 cells. Left panel: representative dot plots. Right panel: statistics. N=5 per group. ^*^, p<0.05; ^**^, p<0.001; ^***^, p<0.001.

To determine effects of SESN2 and SESN3 *in vivo*, lentivirus-transduced NK-92 were pre-treated with doxycycline and transferred into mouse peritoneum. Two days later, NK-92 cells were isolated from the peritoneal cavity based on CD56 expression (Figure 5A). The proportions of SESN2 or SESN3-overexpressing NK-92 cells in total peritoneal cells were similar to the proportions of NK-92 cells not overexpressing SESNs (Figure 5B). Additionally, SESN2 or SESN3-overexpressing NK-92 cells showed more phosphorylated AMPKαand less phosphorylated mTOR, as compared with other groups (Figure 5C & 5D). Moreover, granzyme B expression was significantly lower in SESN2 or SESN3-overexpressing NK-92 cells (Figure 5E). Perforin expression was not altered (Figure 5E).

### SESN2 and SESN3 inhibits tumoricidal effect of NK-92 cells *in vivo*

**Figure 6:**
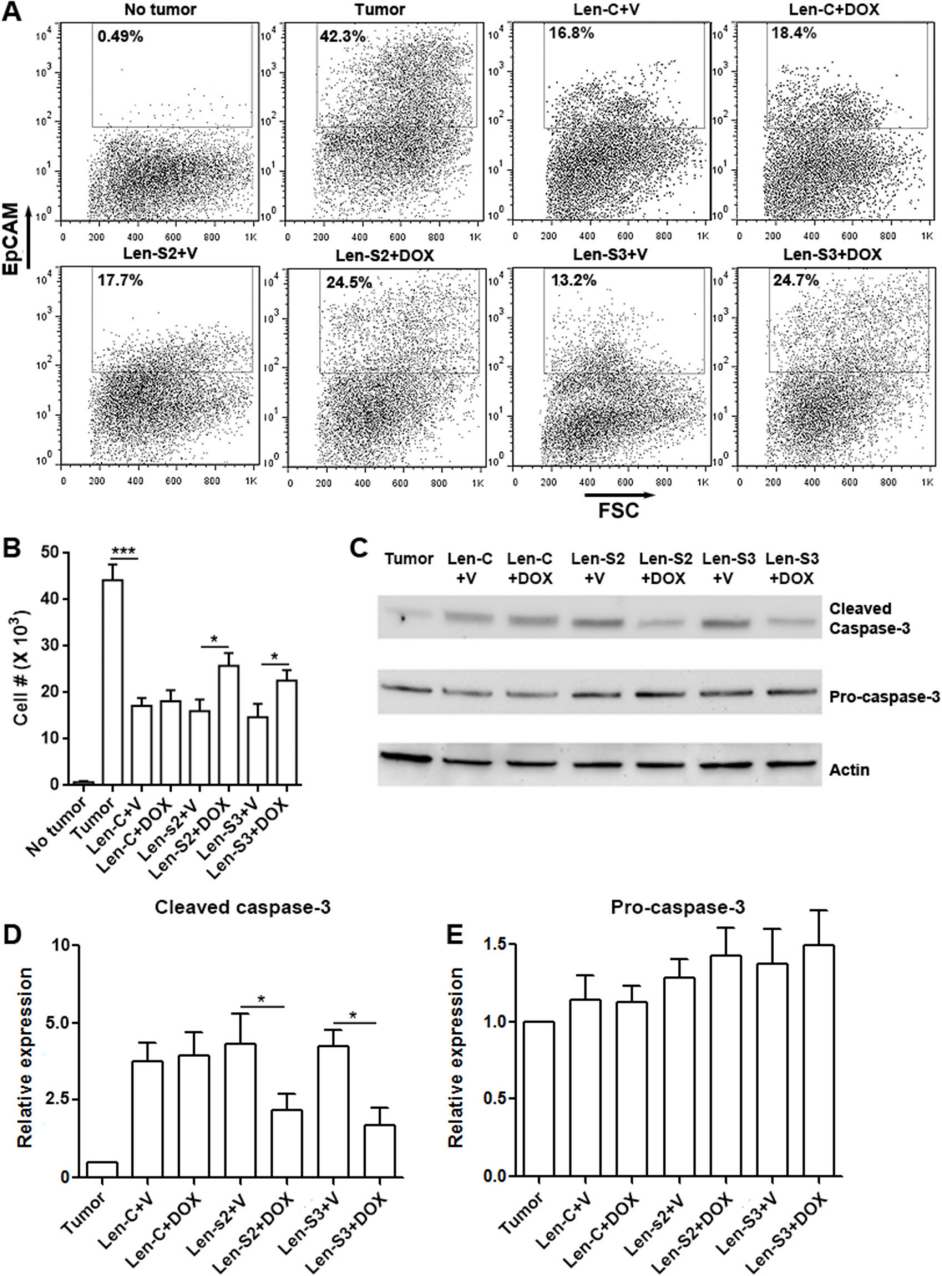
SESN2 and SESN3 suppresses NK-92 cell-mediated anti-ovarian cancer response *in vivo* **(A)** Representative dot plots showing the proportions of EpCAM-expressing OVCAR-3 cells in peritoneal cells. No tumor: no tumor inoculation. Tumor: peritoneal tumor inoculation. Len-S2+V: transfer of Len-S2-transduced NK-92 cells with peritoneal PBS injections. Len-S2+DOX: transfer of Len-S2-transduced NK-92 cells with peritoneal doxycycline injections. Len-S3+V: transfer of Len-S3-transduced NK-92 cells with peritoneal PBS injections. Len-S3+DOX: transfer of Len-S3-transduced NK-92 cells with peritoneal doxycycline injections. **(B)** Absolute number of EpCAM-expressing OVCAR-3 cells harvested from mouse peritoneal cavity. N=7 per group. **(C** to **E)** Activation of caspase-3 in EpCAM-expressing OVCAR-3 cells isolated from mouse peritoneal cavity. Representative Western blot images were shown in (C). Statistics for cleaved caspase-3 was shown in (D). Statistics for pro-caspase-3 expression was shown in (E). N=3 per group. ^*^, p<0.05; ^***^, p<0.001.

To evaluate the tumoricidal activity of NK-92 cells *in vivo*, we conducted a peritoneal OVCAR-3 xenograft model based on a previous study [27]. OVCAR-3 cells were inoculated in the peritoneal cavity of NOD/SCID/γc^−/−^ mice which lack mature NK cells. NK-92 cells, pre-treated with or without doxycycline, were injected into the peritoneal cavity 2 days after inoculation. Four days later, peritoneal NK-92 cells and OVCAR-3 cells were analyzed. As shown in Figure 6A & 6B, in comparison with mice not receiving NK-92 cells, mice receiving vehicle-treated NK-92 cells had less EpCAM^+^ OVCAR-3 cells, suggesting that NK-92 cells killed OVCAR-3 cells *in vivo*. However, in mice receiving doxycycline-treated NK-92 cells, the OVCAR-3 cell abundance was partially restored. Analysis of caspase-3, which is an apoptosis marker, showed less cleaved caspase-3 in OVCAR-3 cells isolated from mice receiving doxycycline-treated NK-92 cells (Figure 6C to 6E). Therefore, the tumoricidal activity of SESN2 or SESN3-overexpressing NK-92 cells was impaired *in vivo*.

## DISCUSSION

SESNs are a family of highly conserved proteins that are induced upon various conditions of stress, including DNA damage and oxidative stress [12]. Physiologically, the activity of SESNs is modulated by nutrients including amino acids at both transcriptional and post-transcriptional levels. SESNs suppress oxidative damage through activation of Nrf2, autophagic removal of impaired mitochondria, and detoxification of reactive oxygen species via its peroxidase activity [12].

However, the significance of SESNs for immune cell development and functions have not been fully elucidated, probably due to their low expression levels in normal immune cells and organs. In this study we identified up-regulation of SESN2 and SESN3 in intratumoral NK-92 cells, suggesting that the tumor microenvironment induced the expression of SESN2 and SESN3. However, the factors that induced SESN2 and SESN3 expression remain unclear. A previous research shows that NO and hypoxia up-regulate SESN2 in macrophages [28]. Indeed, hypoxic stress inside the tumor microenvironment impairs NK cell cytotoxicity [29], probably through inhibiting expression of activating receptors, granzyme B and perforin [30, 31]. Other factors such as mitochondria-dependent ROS production [32] and Toll-like receptor signaling [33] might also induce SESN expression. Interestingly, CD56, CD16 and CD158b are differentially expressed on NK cells in tumor-involved lymph nodes and uninvolved lymph nodes [34]. It will be necessary to check if their expression are intertwined with SESN expression.

The lentiviral system we used is a tetracycline inducible expression system in which SESN expression was induced by doxycycline, therefore avoiding immediate phenotypic and functional alterations of NK-92 cells after lentiviral transduction. We revealed that SESN2 and SESN3 suppressed tumoricidal activity of NK-92 cells. Notably, SESN2 or SESN3-induced phenotypic and functional changes of NK-92 cells were consistent with previously reported hypoxia-mediated changes of NK cells [30, 31]. Therefore, our data suggests the role of hypoxia in the up-regulation of SESNs. Interestingly, SESN3 seemed to be more potent in inhibiting NK-92 cell activity, suggesting that SESN3 might be more active than SESN2. In addition, whether SESN2 and SESN3 have mutual effects on one another needs to be studied in future. Moreover, it will be interesting to check whether normal NK cells up-regulate SESN expression in patients with ovarian cancer or other cancers.

The positive role of mTOR signaling, and the negative role of AMPK signaling in NK cell maturation and activation have been reported [35–37]. The effects of SESNs on mTOR and AMPK signaling have been disclosed in recent studies. SESNs inhibit mTORC1 kinase activation through direct interaction with GATOR2 [16], or through activation of AMPK and TSC [38]. Consistently, here we showed that SESNs-mediated AMPK signaling suppressed mTORC1 signaling in NK-92 cells. Interestingly, the high level of activated AMPKβ1 in SESN-overexpressing NK-92 cells resulted from up-regulation of AMPKβ1 protein, rather than up-regulation of phosphorylation. This data raises the possibility that SESNs might enhance AMPKβ1 expression. Indeed, it has been reported that SESN2 increases mRNA levels of AMPKα1, β1 and γ1 in breast cancer cells [38].

In conclusion, our data highlights the negative effects of SESN2 and SESN3 on NK-92 cell-mediated anti-ovarian cancer activity. Downregulating the expression of SESNs could be beneficial for NK-92 cell-based therapy against ovarian cancer.

## MATERIALS AND METHODS

### Cells

NK-92 cells and OVCAR-3 cells (ovarian cancer cell line) were purchased from Shanghai Baili Biotechnology Co., Ltd (Shanghai, China). NK-92 cells were cultured in α-MEM (Gibco, Gaithersburg, MD, USA) with 10% fetal bovine serum (HyClone Laboratories, Logan, UT, USA), 100 U/ml penicillin/streptomycin (Gibco), and 100 U/ml recombinant hIL-2 (R&D Systems, Minneapolis, MN, USA). OVCAR-3 cells were cultured in RPMI-1640 Medium (Gibco) with 20% fetal bovine serum and 100 U/ml penicillin/streptomycin. Cells were subcultured every 2 days.

### Tumor xenograft models and NK-92 cell adoptive transfer

Animal tests were approved by the Fujian Medical University Animal Care and Use Committee, and were conducted in accordance with institutional guidelines for animal use. Eight week old NOD/SCID/γc^−/−^ mice (C57BL/6J background) were purchased from Beijing Vitalstar Biotechnology (Beijing, China). The subcutaneous tumor xenograft model was established by subcutaneous inoculation of 1 × 10^6^ OVCAR-3 cells on the left flank of each mouse. Thirty days later, NK-92 cells (1 × 10^7^ cells in 100 μl of PBS) were injected into the peritoneum once a day for 2 days. Each mouse then received intraperitoneal injections of recombinant hIL-2 (5 μg per mouse per day) for 6 days. At day 37 after inoculation, peritoneal cells were collected by rinsing the peritoneal cavity with 1 ml of PBS. Spleens and tumor xenografts were mechanically dissociated on 70-μm cell strainers to prepare single cell suspensions.

The intraperitoneal tumor xenograft model was conducted following previous reports with minor modifications [27, 39]. Briefly, mice were given 5 × 10^5^ OVCAR-3 cells (in 50 μl of PBS) via intraperitoneal injection 2 days prior to NK-92 cell injection. NK-92 cells (5 × 10^6^ cells per mouse in 100 μl of PBS) were given to mice via intraperitoneal injection. Mice received hIL-2 as described above. Mice without NK-92 cell injection also received hIL-2. At day 6 after OVCAR-3 cell injection, cells in the peritoneal cavity were collected the same way as above.

### Flow cytometry and cells sorting

The following antibodies were purchased from Biolegend (San Diego, California, USA): APC anti-CD45 (2D1), PE anti-CD56 (39D5), APC anti-EpCAM (9C4), Alexa Fluor® 647 anti-granzyme B (GB11), PE/Cy7 anti-Perforin (B-D48), Alexa Fluor® 647 anti-NKp44 (P44-8), PE anti-NKG2D (1D11), PE anti-NKp46 (9E2), PE anti-NKp30 (P30-15). Rabbit polyclonal anti-phospho-AMPKα (T183/T172) was purchased from Abcam (Cambridge, United Kingdom). PE/Cy7 anti-phospho-mTOR (S2448, clone# MRRBY) was purchased from eBioscience (San Diego, California, USA). Alexa Fluor® 647 polyclonal goat anti-rabbit IgG was purchased from Thermo Fisher (Shanghai, China). For cell surface marker staining, cells were incubated with 2 μg/ml antibody in PBS on ice for 15 min. For cytokine detection, 10 μg/mL brefeldin A (Sigma-Aldrich, St. Louis, MO, USA) was added 3 hours before harvesting cells. Cells were then fixed with 4% paraformaldehyde for 15 minutes, permeabilized with 0.1% Triton™ X-100 for 20 minutes, and incubated with 5 μg/ml cytokine antibodies for 1 h at room temperature. Cell apoptosis was analyzed using APC Annexin-V (eBioscience) following the vendor's manual. Apoptotic cells were identified as Annexin V-positive cells. Cells were analyzed on a BD LSRII flow cytometer. Cell sorting was performed on a BD Influx™ cell sorter.

### Lentiviral transduction

Plasmids pRK5-FLAG-SESN2, pRK5-FLAG-SESN3 and pCAG-GFP were purchased from Addgene (Cambridge, Massachusetts, USA). pLVX-TRE3G-IRES vector was purchased from Shanghai TranSheepBio Co., Ltd (Shanghai, China). The following primers were used to amplify human SESN2 or SESN3 coding sequences and EGFP sequence from these plasmids: SESN2 (5’-ggatccatgatcgtggcggactccgagtg-3’ and 5’-gcggccgctcaggtcatgtagcgggtgatgg-3’); SESN3 (5’-ggatccatgaaccggggcggcggcagc-3’ and 5’-gcggccgctcaggtcaaatgccgagttatgg-3’); EGFP (5’-cccgggatggtgagcaagggcgagg-3’ and 5’-gaattccttgtacagctcgtccatgc-3’). Platinum™ Taq DNA Polymerase (Thermo Fisher) was used for PCR. SESN2 or SESN3 coding sequences were inserted into pLVX-TRE3G-IRES vector via digestion with restriction endonucleases BamHI and NotI, followed by ligation with T7 DNA Ligase. The new pLVX-TRE3G-SESN2-IRES and pLVX-TRE3G-SESN3-IRES vectors were further ligated with EGFP sequence after digestion with SmaI and EcoRI. The resultant vectors were designated pLVX-TRE3G-SESN2-IRES-EGFP and pLVX-TRE3G-SESN3-IRES-EGFP.

For packaging lentiviruses, 2.5×10^6^ HEK293 cells were cultured with 25 μM chloroquine diphosphate (Sigma-Aldrich). 0.72 pmol pRSV-Rev, 1.3 pmol pMDLg/pRRE (both from Addgene), 1.64 pmol pLVX-TRE3G-SESN2-IRES-EGFP or pLVX-TRE3G-SESN3-IRES-EGFP were mixed with 83.4 μg of PEI in 1 ml of Opti-MEM (Gibco). The mixture was then added to HEK293 cells for 18 hours before refreshing the medium. The viral supernatant was harvested 48 and 72 hours later. Lentivirus containing SESN2 or SESN3 sequence was designated Len-S2 and Len-S3, respectively. Lentivirus prepared using empty pLVX-TRE3G-IRES vector was designated Len-C. Packaging of lentivirus containing rtTA3 sequence (Termed Len-rtTA3) was conducted the same way as above, except that pLenti CMV rtTA3 Blast vector (Addgene) was used.

To transduce NK-92 cells, 1.0 × 10^5^ /ml NK-92 cells were stimulated with 100 U/ml hIL-2 and 100 ng/ml hIL-12 (Both from R&D Systems) for 2 hours. Lentiviral particles were added to cells (Multiplicity of Infection was 30) with 8 μg/ml polybrene (Sigma-Aldrich) for 18 hours. Cells were then selected with 2 μg/ml puromycin (Sigma-Aldrich) for 6 days. Surviving cells were transduced with Len-rtTA3 in the same way, and were selected for additional 6 days with 2 μg/ml blasticidin (Sigma-Aldrich). Surviving cells were expanded for further experiments. To induce overexpression of exogenous genes *in vitro*, cells were treated with 200 ng/ml doxycycline (Sigma-Aldrich) for 48 h. To induce expression *in vivo*, each mouse was intraperitoneally injected with 1 mg/kg doxycycline every two days.

### Cytotoxicity assay

Lenitvirus-transduced NK-92 cells were pre-treated with or without 200 ng/ml doxycycline for 48 h. These cells were then co-cultured with OVCAR-3 cells for 4 h at 37°C (effector : target = 10:1). Cell lysis was determined using LDH Cytotoxicity Assay Kit (Thermo Fisher), because LDH release is widely used in cytotoxicity assay [40]. OVCAR-3 apoptosis was distinguished by staining the whole cells with PE anti-CD56 antibody and APC Annexin-V, because OVCAR-3 cells do not express CD56. In some experiments, during the last 4 hours of doxycycline treatment, NK-92 cells were treated with10 μM compound C for 4 hours before co-culture.

### RNA isolation, reverse transcription and real-time quantitative PCR (q-PCR)

RNA was isolated using the Eastep RNA Extraction Kit (Promega, Beijing, China). mRNA was transcribed into cDNA using VigoScript cDNA Synthesis Kit (Vigorous Biotechnology, Shanghai, China). q-PCR was performed using SYBR® Green Master Mix (Thermo Fisher) on a 7300 thermocycler (Invitrogen, Carlsbad, CA, USA). Primer sequences are as follows: TNF-α: 5'-ggagaagggtgaccgactca-3' and 5'-ctgcccagactcggcaa-3'. β-actin: 5'-tcacccacactgtgcccatctacg-3' and 5'-cagcggaaccgctcattgccaatg-3'.

### Western blot

Proteins were extracted using RIPA buffer (Thermo Fisher). The following antibodies were used: anti-β-actin-actin (sc-47778), anti-SESN2 (sc-101249), anti-phospho-mTOR (S2448, sc-293133), anti-mTOR (sc-136269), anti-phospho-4EBP1 (S65, sc-293124), anti-4EBP1 (sc-9977), anti-AMPKα1/2 (sc-74461), anti-AMPKβ1 (sc-100357) and anti-caspase-3 (sc-7272) were purchased from Santa Cruz Biotechnology (Dallas, USA). Anti-SESN1 (ab134091), anti-SESN3 (ab97792), anti-phospho-AMPKα1/2 (T183/T172, ab23875) and anti-phospho-AMPKβ1 (S181, ab55311) were purchased from Abcam. Cleaved caspase-3 (Asp175, MAB835) was purchased from R&D Systems.

### Statistics

Data were presented as mean ± SD. All experiments were independently repeated for at least three times. Data was analyzed by one-way ANOVA followed by Fisher PLSD post hoc tests, or Student's t test. *P* values < 0.05 were considered significant.

## SUPPLEMENTARY MATERIALS FIGURES


