# Supplementary material for: Sestrin2 and sestrin3 suppress NK-92 cell-mediated cytotoxic activity on ovarian cancer cells through AMPK and mTORC1 signaling
Source: Oncotarget. 2017 Oct 4;8(52):90132–43. doi: 10.18632/oncotarget.21487 (PMC5685737; doi:10.18632/oncotarget.21487)
Supplement: Supplementary file 1 [file oncotarget-08-90132-s001.pdf]

Sestrin2 and sestrin3 suppress NK-92 cell-mediated cytotoxic activity on ovarian cancer cells through AMPK and mTORC1 signaling

SUPPLEMENTARY MATERIALS

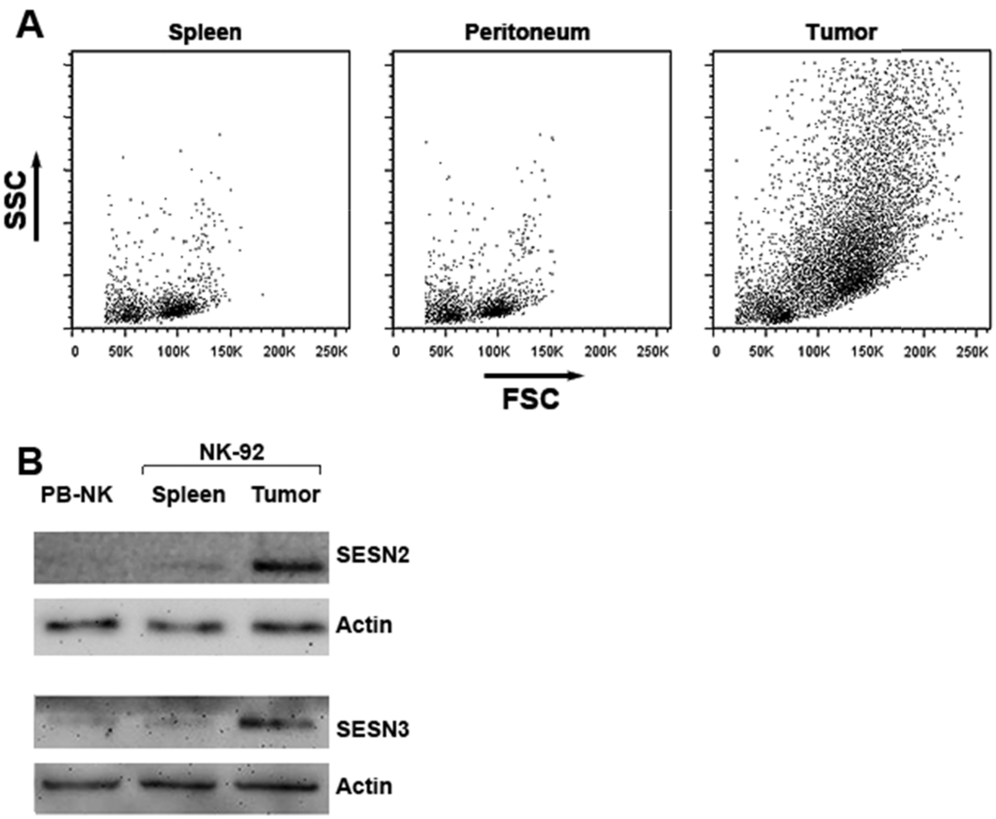

Supplementary Figure 1: (A) Representative dot plots of the whole cells harvested from tumor-bearing mouse spleens, peritoneum and tumor xenografts. **(B)** Expression of SESN2 and SESN3 in human peripheral blood $CD56^+$ NK cells (PB-NK) and transferred NK-92 cells isolated from mouse spleens and OVCAR3 xenografts, respectively. Peripheral blood NK cells were sorted from healthy donors by flow cytometry. This is a representative image of two independent experiments.

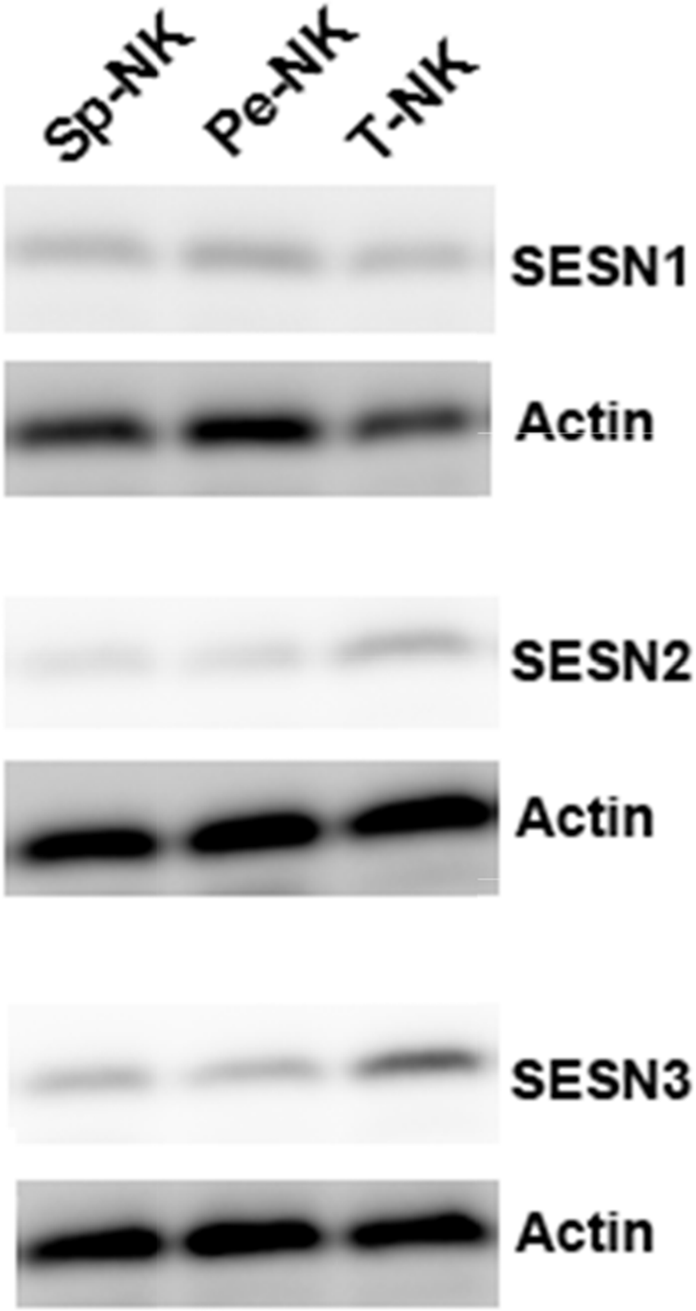

Supplementary Figure 2: SESN1, SESN2 and SESN3 protein levels in NK-92 cells in mouse bearing SKOV3 tumor xenografts. Sp-NK: NK-92 cells isolated from spleens. Pe-NK: NK-92 cells isolated from peritoneum. T-NK: intratumoral NK-92 cells. This is a representative data of two independent experiments.

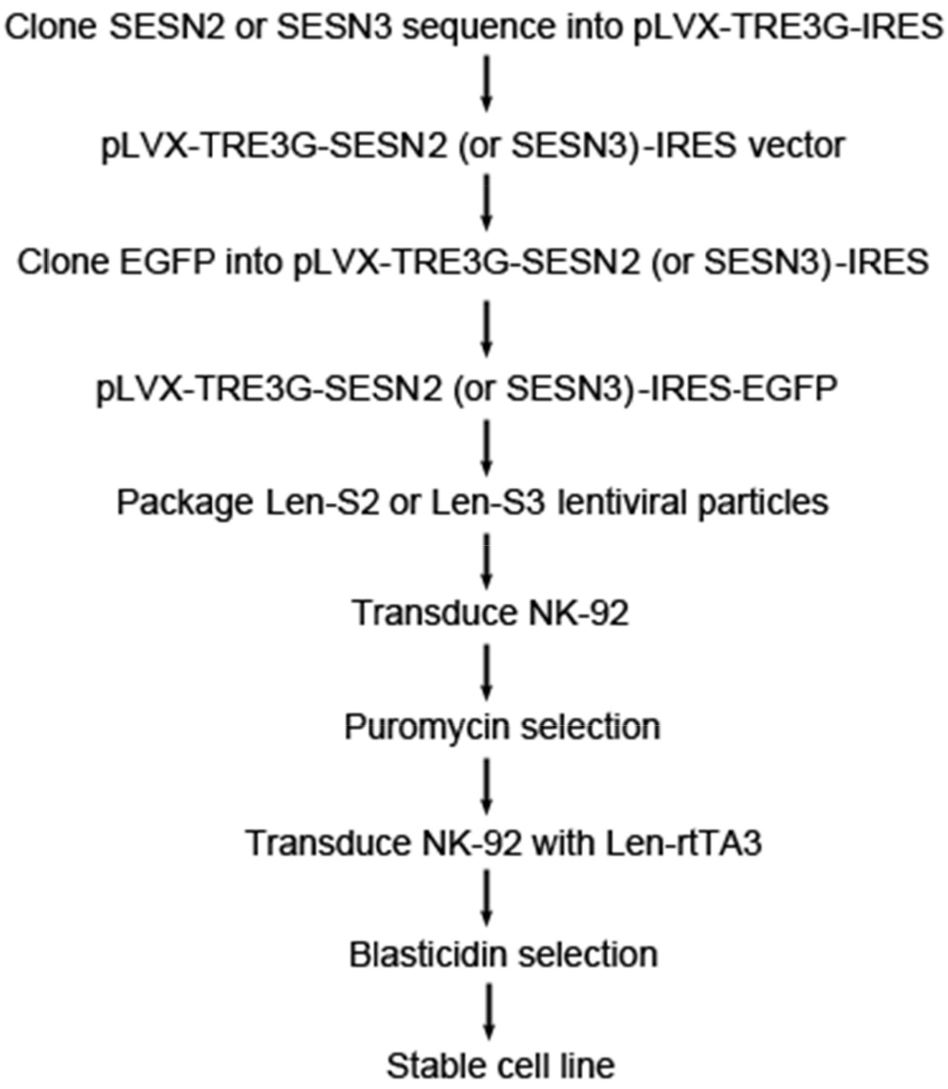

Supplementary Figure 3: Establishment of NK-92 cells expressing inducible SESN2 or SESN3. Len-S2: lentivirus containing SESN2 coding sequence. Len-S3: lentivirus containing SESN3 coding sequence.

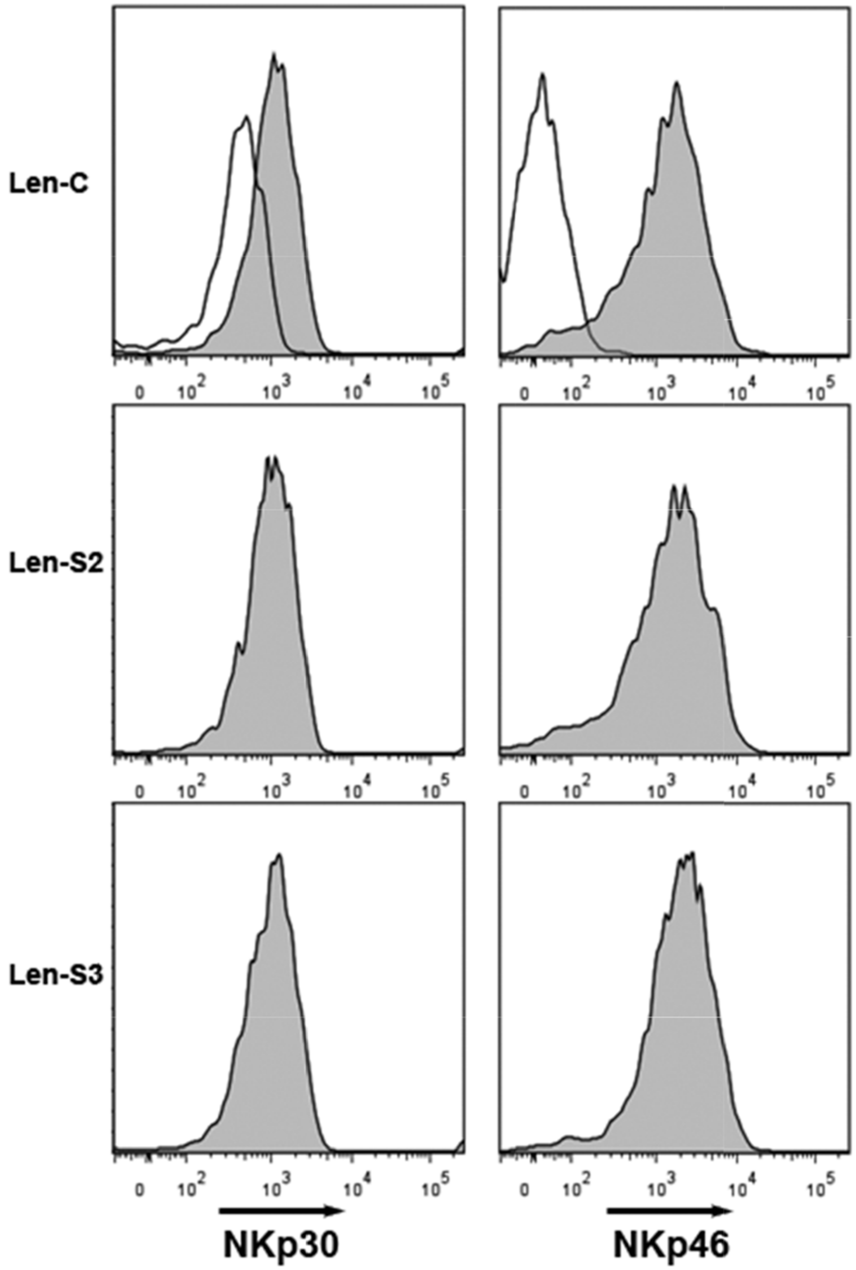

Supplementary Figure 4: Expression of NKp30 and NKp44 on lentivirus-transduced NK-92 cells 48 hours after doxycycline treatment. White curves: isotype control. Grey curves: NKp30 or NKp44 antibody staining.

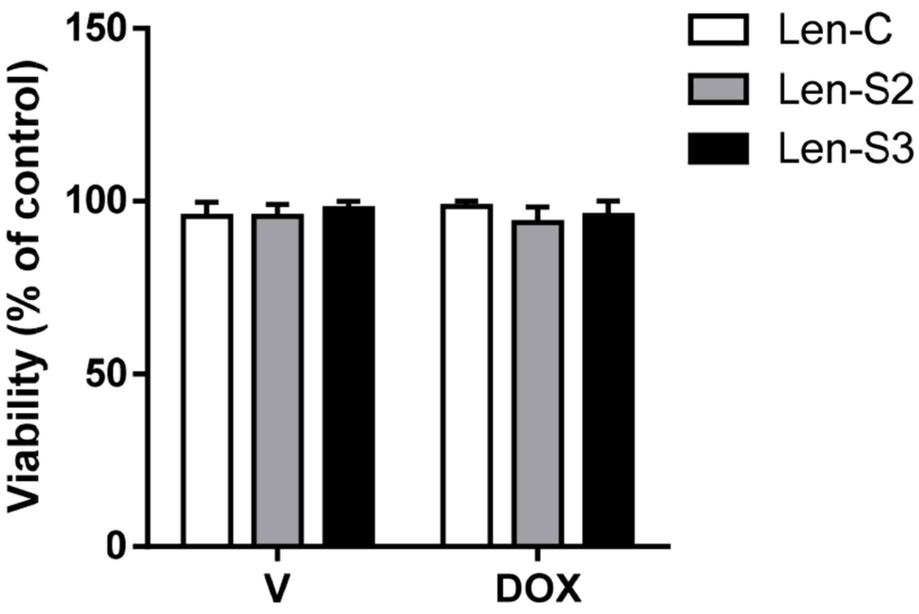

Supplementary Figure 5: Cell viability assay using Calcein-AM. NK-92 cells transduced with Len-C, Lne-S2 or Len-S3 were seeded at a density of 1×10^6/ml cells and were incubated with vehicle or 200 ng/ml doxycycline for 48 h. After that, cells were washed with PBS once, and were then incubated in PBS containing 1 μM Calcein-AM (Sigma-Aldrich) for 10 min at room temperature. The fluorescence was measured using a Synergy™ HTX Multi-Mode Microplate Reader. V: vehicle; DOX: doxycycline. N=6 per group.

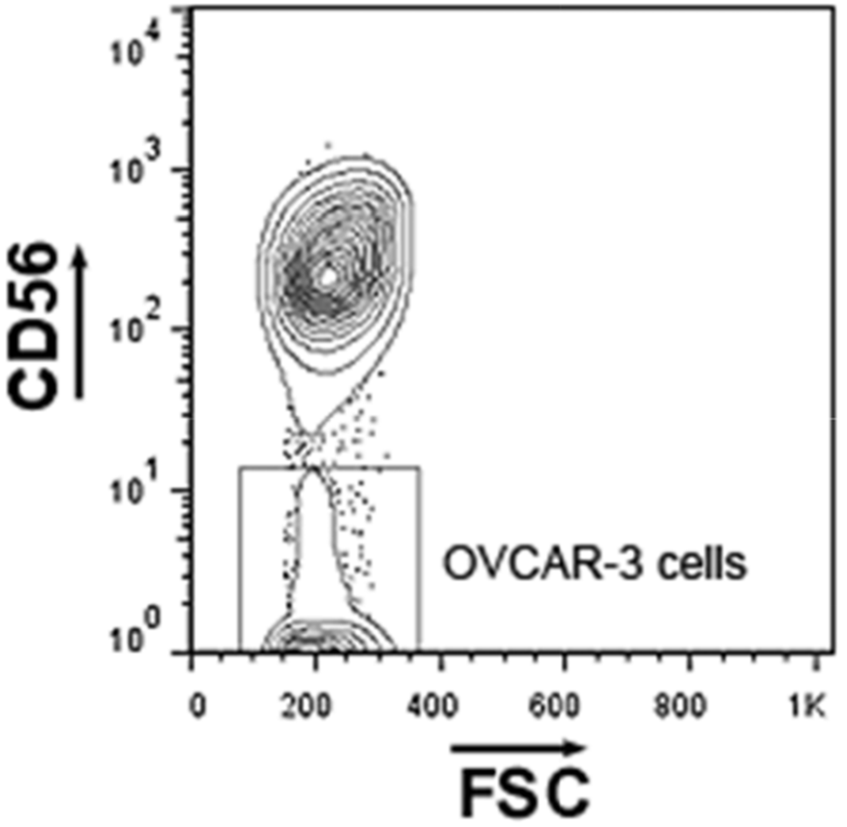

Supplementary Figure 6: OVCAR-3 cells and NK-92 cells were distinguished by CD56 staining after co-culture.

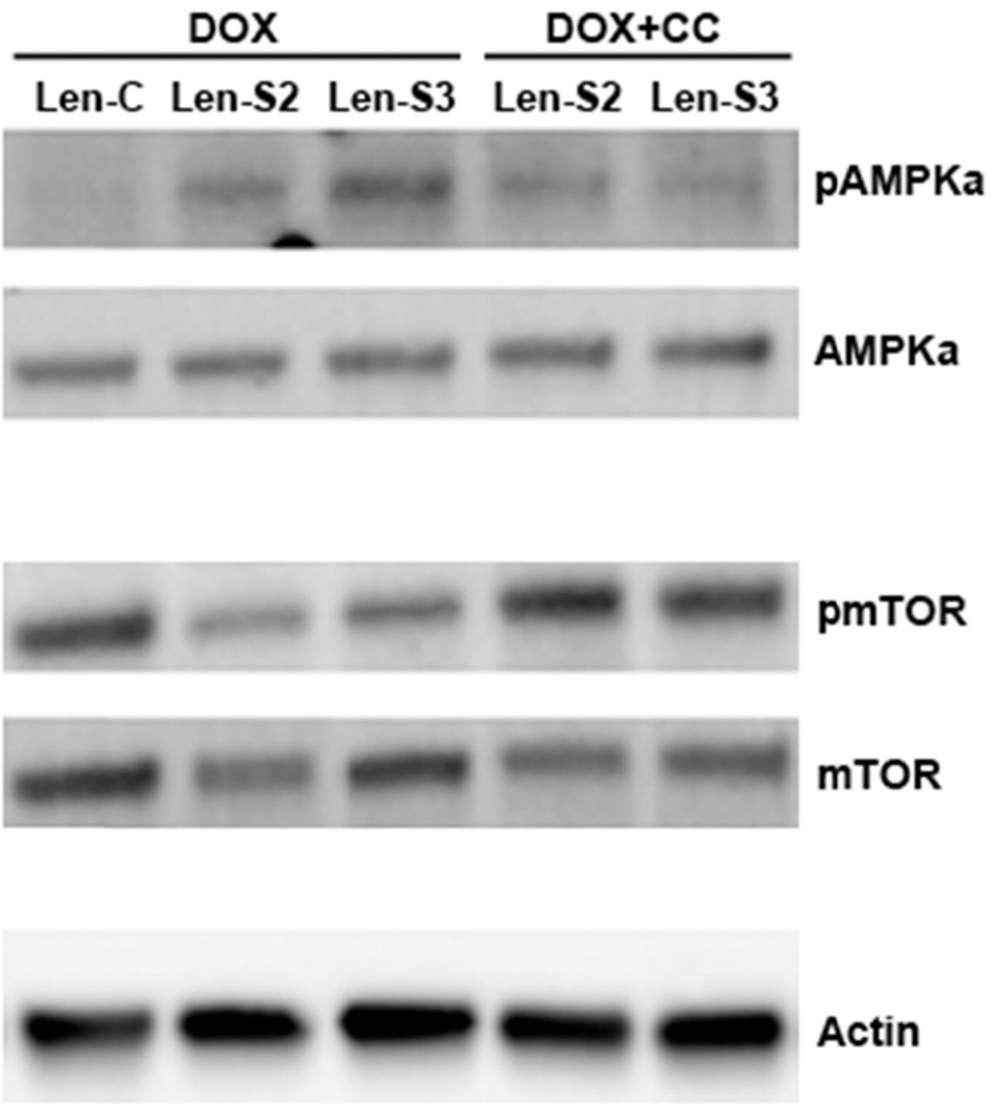

Supplementary Figure 7: Compound C treatment inhibits SESN2 or SESN3-induced AMPKα activation and promotes activation of mTOR phosphorylation. DOX: doxycycline. DOX+CC: doxycycline plus compound C. Len-C: NK-92 cells transduced with lentivirus containing no SESN sequence. Len-S2: NK-92 cells transduced with Len-S2. Len-S3: NK-92 cells transduced with Len-S3. This is a representative data of two independent experiments.
